# Cytochrome P450-epoxygenated fatty acids inhibit Müller glial inflammation

**DOI:** 10.1038/s41598-021-89000-1

**Published:** 2021-05-06

**Authors:** Cayla D. Ontko, Megan E. Capozzi, Minjae J. Kim, Gary W. McCollum, John S. Penn

**Affiliations:** 1 Department of Molecular Physiology and Biophysics, Vanderbilt University School of Medicine, Nashville, TN USA; 2 Duke Molecular Physiology Institute, Duke University, Durham, NC USA; 3 Department of Ophthalmology and Visual Sciences, Vanderbilt University Medical Center, Nashville, TN USA

**Keywords:** Drug development, Preclinical research, Translational research, Retinal diseases, Diabetes complications

## Abstract

Free fatty acid dysregulation in diabetics may elicit the release of inflammatory cytokines from Müller cells (MC), promoting the onset and progression of diabetic retinopathy (DR). Palmitic acid (PA) is elevated in the sera of diabetics and stimulates the production of the DR-relevant cytokines by MC, including IL-1β, which induces the production of itself and other inflammatory cytokines in the retina as well. In this study we propose that experimental elevation of cytochrome P450 epoxygenase (CYP)-derived epoxygenated fatty acids, epoxyeicosatrienoic acid (EET) and epoxydocosapentaenoic acid (EDP), will reduce PA- and IL-1β-induced MC inflammation. Broad-spectrum CYP inhibition by SKF-525a increased MC expression of inflammatory cytokines. Exogenous 11,12-EET and 19,20-EDP significantly decreased PA- and IL-1β-induced MC expression of IL-1β and IL-6. Both epoxygenated fatty acids significantly decreased IL-8 expression in IL-1β-induced MC and TNFα in PA-induced MC. Interestingly, 11,12-EET and 19,20-EDP significantly increased TNFα in IL-1β-treated MC. GSK2256294, a soluble epoxide hydrolase (sEH) inhibitor, significantly reduced PA- and IL-1β-stimulated MC cytokine expression. 11,12-EET and 19,20-EDP were also found to decrease PA- and IL-1β-induced NFκB-dependent transcriptional activity. These data suggest that experimental elevation of 11,12-EET and 19,20-EDP decreases MC inflammation in part by blocking NFκB-dependent transcription and may represent a viable therapeutic strategy for inhibition of early retinal inflammation in DR.

## Introduction

Diabetic retinopathy (DR) is the leading cause of irreversible vision loss among working age Americans, affecting ~ 35% of patients with diabetes mellitus^1^. As the prevalence of worldwide diabetes increases, the number of people suffering from diabetes-induced vision loss increases as well^2^. DR pathology is classified in two clinically distinct forms, non-proliferative (NPDR) and proliferative (PDR). NPDR is characterized by the appearance of microaneurysms, focal hemorrhaging, hard exudates beneath the retinal surface and retinal capillary death^3,4^. The death of retinal capillaries in NPDR can result in vasoregression-promoted ischemia, causing retinal hypoxia that elicits the synthesis and release of vascular endothelial cell growth factor (VEGF)^5^. Increased levels of retinal VEGF can trigger a vasoproliferative response, transitioning the retina to vision threatening PDR^5^. Current DR therapies, such as laser photocoagulation or VEGF inhibition, target PDR after irreversible retinal damage has occurred. Therefore, there is an important unmet need to develop a therapy that intervenes prior to PDR onset to preserve retinal function.

DR progression is associated with systemic dyslipidemia, and circulating free fatty acids (FFAs) are known to initiate inflammatory cytokine release^6,7^. Diabetic mice have over three times the retinal fatty acid content of healthy controls and palmitic acid (PA) is elevated above other FFAs in the circulation and tissues of diabetic patients and experimental models of diabetes^8–10^. The detrimental effects of FFAs in the diabetic retina has been substantiated in two epidemiological human studies, ACCORD and FIELD, in which the lipid-lowering drug fenofibrate was shown to delay retinopathy progression^6,7^. Müller cells (MC) are particularly responsive to PA and other FFA^8,11^. RNA sequencing has shown that PA stimulates a variety of DR-relevant pathways in MC, including NFκB signaling and inflammation, intracellular lipid signaling, angiogenesis, and MAPK signaling, that are not altered by elevated glucose stimulation alone^8^. It is proposed that diabetes-related dysregulation of PA and other FFAs damage MC, resulting in their production of inflammatory retinal cytokines^8,12^. These cytokines amplify through autocrine and paracrine mechanisms, reaching levels that promote chronic retinal inflammation^5^. If these levels are sustained, retinal vascular pathology can ensue, promoting DR progression. In support of this notion, studies in human patients and animal models show that elevated levels of inflammatory cytokines in the vitreous and retina correlate with early DR progression^5,12–14^. One such cytokine, interleukin 1β (IL-1β), is purported to be a “master regulator” of cytokine-induced inflammation^15,16^. IL-1β is elevated in DR and induces MC to produce and release itself and other inflammatory cytokines. MC are vital to retinal homeostasis and may become activated in response to diabetes-related metabolic dysfunction. This causes a diversion from their homeostatic functions, promoting DR onset and progression. MC activation is easily assayed by glial fibrillary acidic protein immunostaining of retinal cross-sections and it is one of the earliest observable changes in DR^8,17^. The foregoing, along with other MC-dependent behaviors, suggests that therapies targeting MC inflammation in DR could potentially preempt PDR and its vision threatening consequences.

Ample data suggest that lipid mediators derived from ω-6 and ω-3 fatty acids regulate diabetes-induced retinal inflammation^5,18,19^. Arachidonic acid (AA; ω-6) and docosahexaenoic acid (DHA; ω-3) are polyunsaturated fatty acids (PUFAs) found at high abundance in the retina, suggesting their importance in retinal physiology^20–22^. These PUFAs are metabolized through the cyclooxygenase (COX), lipoxygenase (LOX) or cytochrome P450 epoxygenase (CYP) pathways. Although there are exceptions, AA is metabolized by COX and LOX to yield oxygenated metabolites that are largely pro-inflammatory^5,23^. For example, it has been shown that COX inhibitors such as aspirin and other NSAIDs reduce DR associated inflammation^24^. Unlike AA, it has been reported that COX and LOX convert DHA into anti-inflammatory metabolites^23^. COX converts DHA to hydroxyl DHA, and 15-lipoxygenase (ALOX15) converts DHA to 17S-hydroperoxy-DHA that is further metabolized to yield the D-resolvins^25–27^. Streptozotocin-induced diabetic rats that received intravitreal injections of resolvin D1 demonstrated reduced levels of retinal IL-1β and NFκB activity^26^. There is growing interest in the epoxygenation of ω-6 and ω-3 fatty acids by cytochrome P450 epoxygenases (CYPs). CYPs are endoplasmic reticulum membrane-bound monooxygenases that metabolize fatty acids to epoxide derivatives that demonstrate potent anti-inflammatory activities in a variety of biological systems^3^. CYP2C8, CYP2C9, and CYP2J2 are the most well-characterized human CYPs that epoxygenate AA and DHA to yield epoxyeicosatrienoic acids (EET) and epoxydocosapentaenoic acids (EDP), respectively^3,19^. AA yields four regioisomers, 5,6-EET, 8,9-EET, 11,12-EET, and 14,15-EET, while DHA yields six regioisomers, 4,5-EDP, 7,8-EDP, 10,11-EDP, 13,14-EDP, 16,17-EDP, and 19,20-EDP^3^. We have previously shown that the administration of exogenous 11,12-EET reduces the expression of the leukocyte adhesion protein VCAM1 in human retinal microvascular endothelial cells (hRMEC) activated by TNFα^3^. These data suggest that increasing EET/EDP levels may be an effective method to reduce DR-related inflammation.

Soluble epoxide hydrolase (sEH) hydrolyzes EET and EDP to their less biologically active diols, dihydroxyeicosatrienoic acid (DHET) and dihydroxydocosapentaenoic acid (DHDP)^3^. By reducing the half-life of epoxides, sEH decreases their abundance in tissues and thus the potency of their anti-inflammatory activities. Therefore, sEH inhibition presents a rational therapeutic method to elevate epoxide levels and reduce inflammation. sEH inhibitors (sEHi) have been tested in animal models of inflammatory disease to raise EET/EDP levels and mitigate inflammation^28^. These successful studies have led to clinical trials testing sEH inhibition in diabetes-relevant pathologies, such as impaired glucose tolerance and insulin resistance^30^. Furthermore, studies that use sEH inhibitors in combination with other pharmacologic strategies to raise EET/EDP levels prove more efficacious than sEHi’s administered alone^3^. For example, TNFα-induced leukocyte adhesion expression in human retinal endothelial cells was significantly reduced with the administration of sEHi and EET/EDP in combination, but not separately^3^.

CYP levels are suppressed in diabetic conditions, and patients with NPDR and PDR have reduced levels of EETs observed in the vitreous^31,32^. It was also found that soluble epoxide hydrolase activity is increased in response to diabetic conditions, contributing to lower epoxygenated fatty acids levels, and creating conditions permissive to inflammation^33,34^. Thus, pharmacologic manipulations that elevate epoxygenated fatty acids might constitute a rational strategy to reduce retinal inflammation in DR. In this study, we tested the efficacy of increased epoxygenated fatty acid concentrations to mitigate PA- and IL-1β-induced expression of inflammatory cytokines in primary cultures of human Müller cells (hMC). The levels of 11,12-EET and 19,20-EDP were manipulated in hMC cultures via CYP inhibition, exogenous addition of epoxides, and the inhibition of epoxide hydrolysis.

## Results

### The CYP epoxygenase inhibitor SKF-525a promotes inflammatory cytokine expression in hMC

**Figure 1:**
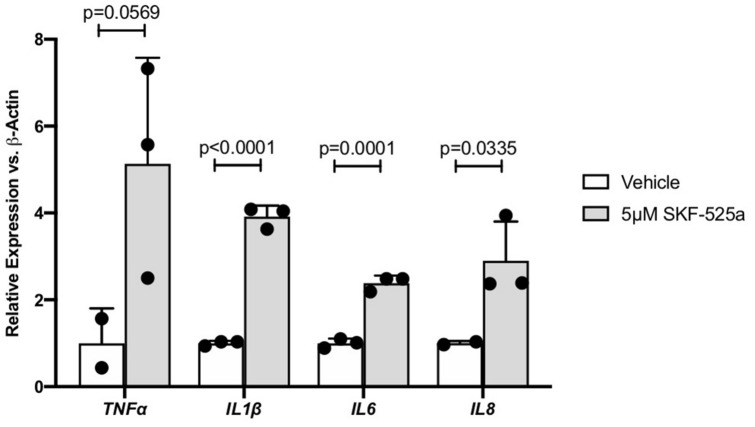
The effect of CYP inhibitor SKF-525a on Müller cell inflammatory cytokine expression. Human Müller cells were treated with SKF-525a (5.0 μM) for 24 h. After treatment, total RNA was isolated and inflammatory cytokine expression was assayed by qRT-PCR. *TNFα*, *IL1β*, *IL6* and *IL8* expression was increased by SKF-525a, though statistical significance was not achieved for *TNFα*. Data are displayed as mean ± SD (n = 2 or 3).

hMC were treated with the CYP epoxygenase inhibitor SKF-525a or vehicle and inflammatory cytokine expression was assayed via qRT-PCR. SKF-525a increased expression of the DR-relevant cytokines *TNFα* (5.13 fold; *p* = 0.0569), *IL1β* (3.92 fold; *p* < 0.0001)*, IL6* (2.38 fold; *p* = 0.0001), and *IL8* (2.90 fold; *p* = 0.0335). Only *TNFα* did not achieve statistical significance (Fig. 1).

### 11,12-EET, 19,20-EDP or the sEH inhibitor GSK2256294 reduces PA-stimulated inflammatory cytokine expression

**Figure 2:**
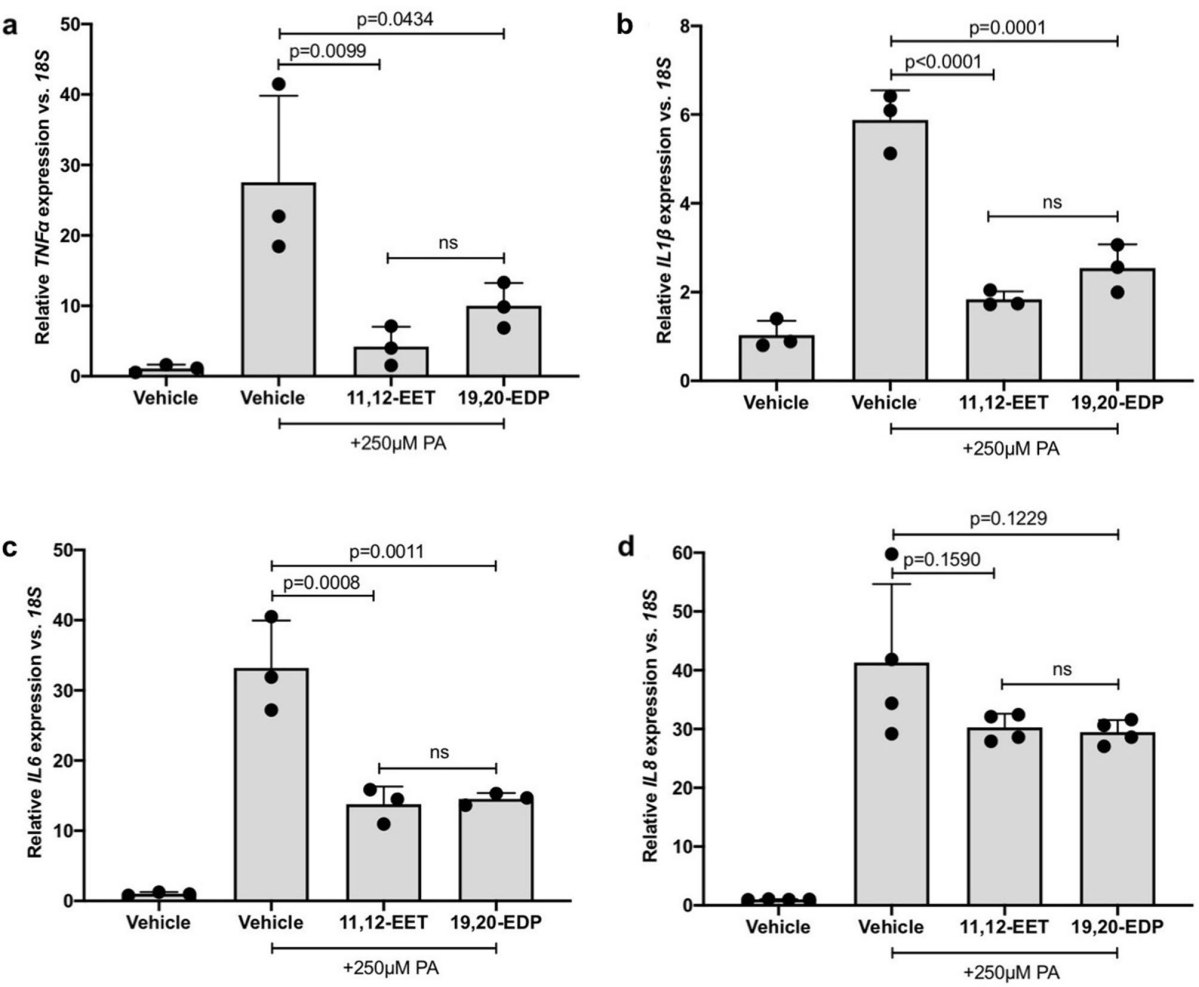
The effect of 11,12-EET and 19,20-EDP on PA-induced inflammatory mediator expression by Müller cells. Human Müller cells were treated with PA (250 μM) for 24 h. 11,12-EET (0.5 μM) or 19,20-EDP (0.5 μM) was added during the final 3 h of treatment. After 24 h, total RNA was isolated and expression was assayed by qRT-PCR. (**a**) *TNFα* (**b**) *IL1β*, and (**c**) *IL6* expression was significantly decreased by both epoxygenated fatty acids. (d) *IL8* expression was reduced but statistical significance was not achieved. Results depicted are representative of three separate experiments. Data are displayed as mean ± SD (n = 3 or 4 for each experiment).

**Figure 3:**
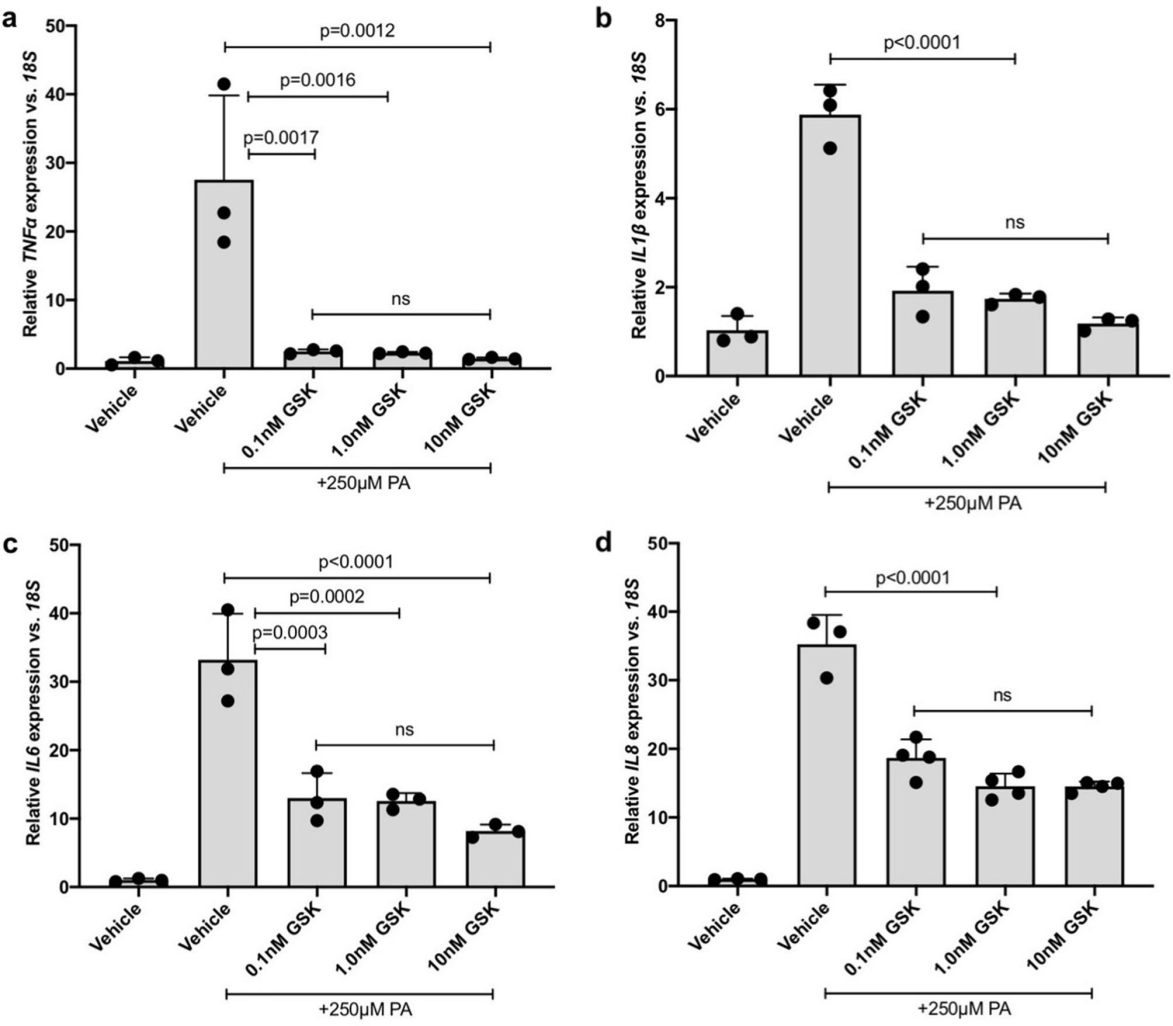
The effect of sEH inhibitor GSK2256294 on PA-induced inflammatory mediator expression by Müller cells. Human Müller cells were treated with PA (250 μM) or PA plus 0.1 nM, 1.0 nM or 10 nM GSK2256294 (sEH inhibitor). After 24 h, total RNA was isolated, and expression was analyzed by qRT-PCR. (**a**) *TNFα*, (**b**) *IL1β*, (**c**) *IL6*, and (**d**) *IL8* expression was significantly decreased by the addition of the sEH inhibitor at each of the concentrations tested. Results depicted are representative of three separate experiments. Data are displayed as mean ± SD (n = 3 or 4 for each experiment).

The epoxygenated fatty acids 11,12-EET, 19,20-EDP or the sEH inhibitor, GSK2256294, were tested against PA-induced inflammatory cytokine expression in hMC. hMC were treated with PA in the presence or absence of 11,12-EET, 19,20-EDP or GSK2256294 and inflammatory cytokine expression was assessed by qRT-PCR. 11,12-EET significantly reduced PA-stimulated expression of *TNFα* by 84.67%, *IL1β* by 68.72% and *IL6* by 58.54% (Fig. 2a–c. *p* = 0.0099, *p* < 0.0001, *p* = 0.0008, respectively). 19,20-EDP significantly reduced PA-stimulated expression of *TNFα* by 63.67%, *IL1β* by 56.76%, and *IL6* by 56.19% (Fig. 2a–c. *p* = 0.0434, *p* = 0.0001, *p* = 0.0011, respectively). 11,12-EET and 19,20-EDP reduced PA-stimulated *IL8* expression by 26.70% and 28.59%, however, statistical significance was not achieved (Fig. 2d). A range of GSK2256294 concentrations were tested (0.1 nM, 1.0 nM, and 10 nM) and GSK2256294 significantly reduced PA-stimulated hMC cytokine expression at each concentration. At the lowest concentration tested, 0.1 nM, GSK2256294 reduced PA-stimulated expression of *TNFα* by 90.94%, *IL1β* by 67.31%, *IL6* by 60.86%, and *IL8* by 47.02% in hMC (Fig. 3a–d. *p* = 0.0017, *p* < 0.0001, *p* = 0.0003, *p* < 0.0001, respectively). At 1 nM, GSK2256294 reduced PA-stimulated expression of *TNFα* by 91.64%, *IL1β* by 70.39%, *IL6* by 62.13%, and *IL8* by 58.74% in hMC (Fig. 3a–d. *p* = 0.0016, *p* < 0.0001, *p* = 0.0002, *p* < 0.0001, respectively). At 10 nM, GSK2256294 reduced PA-stimulated expression of *TNFα* by 94.65%, *IL1β* by 79.87%, *IL6* by 75.36%, and *IL8* by 58.83% in hMC (Fig. 3a–d. *p* = 0.0012, *p* < 0.0001, *p* < 0.0001, *p* < 0.0001, respectively).

### 11,12-EET, 19,20-EDP or the sEH inhibitor GSK2256294 reduces IL-1β-stimulated inflammatory cytokine expression

**Figure 4:**
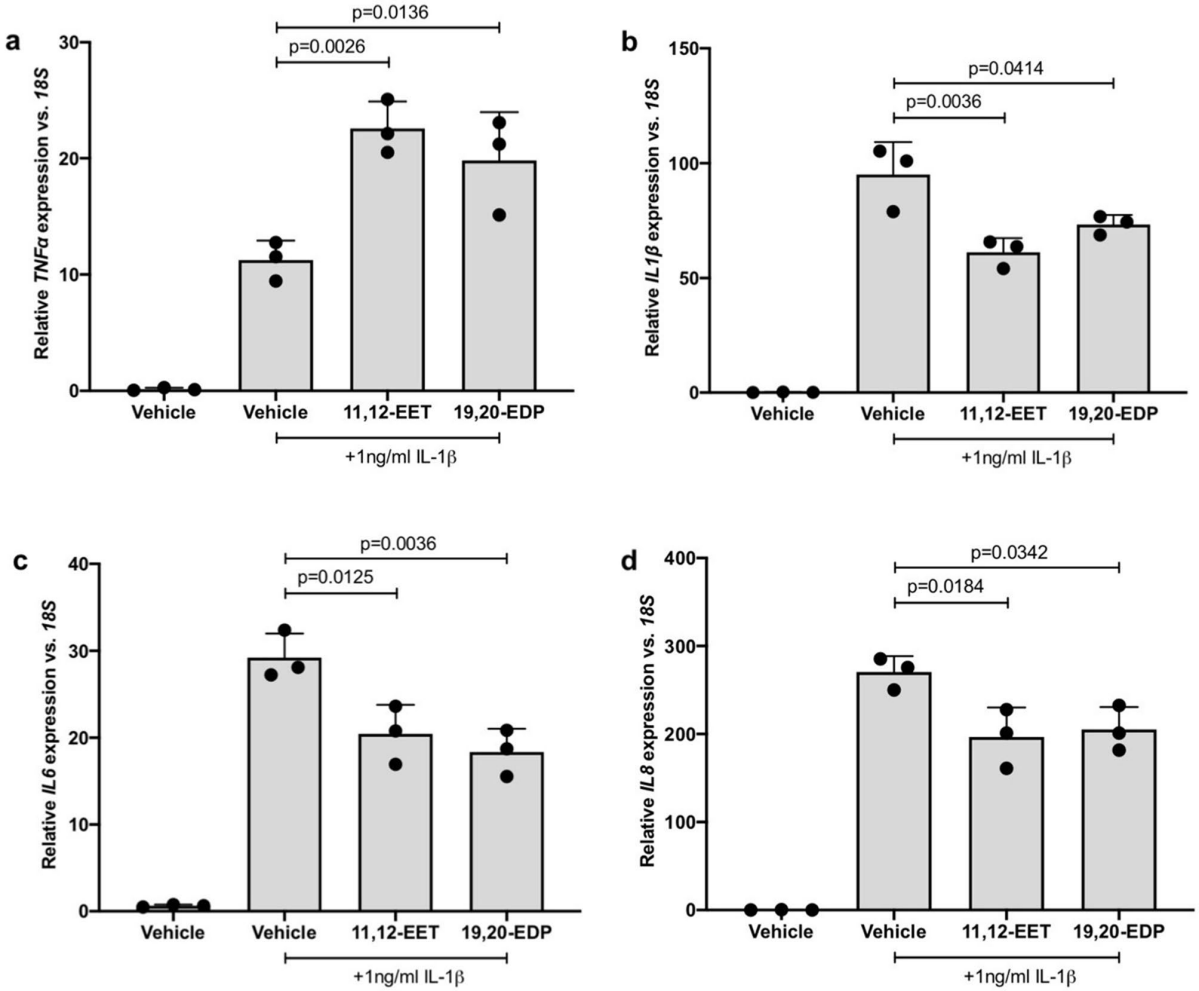
The effect of 11,12-EET and 19,20-EDP on IL-1β-induced inflammatory cytokine expression by Müller cells. Human Müller cells were treated with IL-1β (1.0 ng/ml) or IL-1β plus 11,12-EET (0.5 μM) or 19,20-EDP (0.5 μM) for 8 h. Total RNA was isolated and cytokine expression was assayed by qRT-PCR. (**a**) *TNFα* expression was significantly elevated while (**b**) *IL1β*, (**c**) *IL6*, and (**d**) *IL8* expression was significantly decreased by the addition of both exogenous epoxygenated fatty acids. Results depicted are representative of three separate experiments. These data are normalized to induction levels illustrated in Fig. 5. Data are displayed as mean ± SD (n = 3).

**Figure 5:**
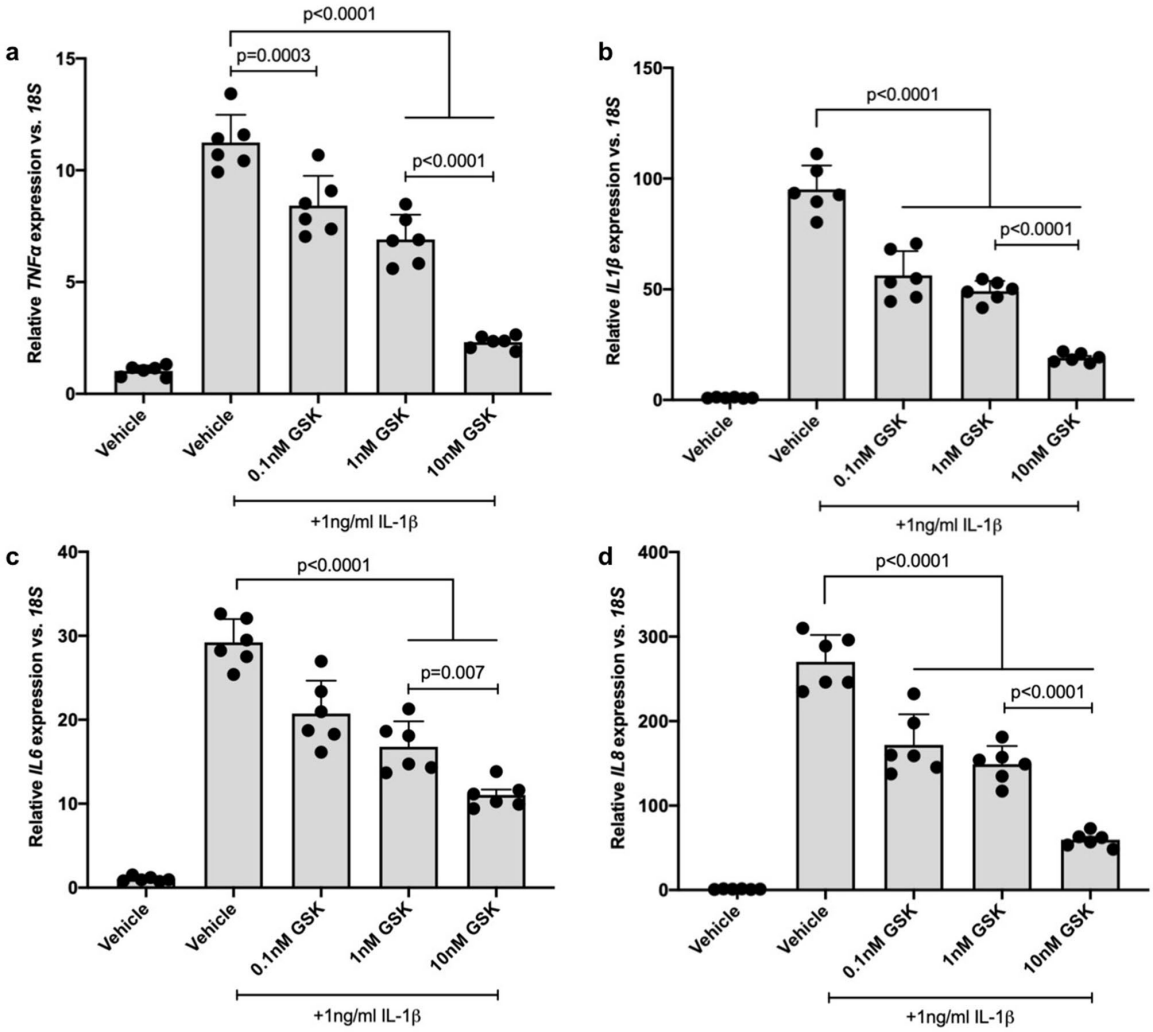
The effect of sEH inhibitor GSK2256294 on IL-1β-induced inflammatory mediator expression by Müller cells. Human Müller cells were treated with IL-1β (1.0 ng/ml) or IL-1β plus 0.1 nM, 1.0 nM, or 10 nM GSK2256294 (sEH inhibitor) for 8 h. Total RNA was isolated and cytokine expression was assayed by qRT-PCR. (**a**) *TNFα,* (**b**) *IL1β*, (**c**) *IL6*, and (**d**) *IL8* expression was significantly decreased at each sEH inhibitor concentration tested. Bars represent mean ± SD (n = 6).

hMC were treated with IL-1β in the presence or absence of 11,12-EET, 19,20-EDP or GSK2256294 to test the effect of each on IL-1β-induced inflammatory cytokine expression. Total RNA was isolated and inflammatory cytokine expression was assessed by qRT-PCR. 11,12-EET significantly reduced IL-1β-stimulated expression of *IL1β* by 35.65%, *IL6* by 30.06%, and *IL8* by 27.26% in hMC (Fig. 4b–d. *p* = 0.0036, *p* = 0.0125, *p* = 0.0184, respectively). 19,20-EDP significantly reduced IL-1β-stimulated expression of *IL1β* by 22.88%, *IL6* by 37.18%, and *IL8* by 24.10% in hMC (Fig. 4b–d. *p* = 0.0414, *p* = 0.0036, *p* = 0.0342, respectively). *TNFα* expression, however, was significantly increased by both epoxygenated fatty acids (Fig. 4a. *p* = 0.0026, *p* = 0.0136). sEH inhibition was tested at a range of GSK2256294 concentrations (0.1 nM, 1.0 nM, and 10 nM) and at each concentration IL-1β-stimulated cytokine expression was significantly reduced. At the lowest concentration tested, 0.1 nM, GSK2256294 reduced IL-1β-stimulated expression of *TNFα* by 25.11%, *IL1β* by 40.78%, *IL6* by 29.05%, and *IL8* by 36.37% in hMC (Fig. 5a–d. *p* = 0.0003, *p* < 0.0001, *p* < 0.0001, *p* < 0.0001, respectively). At 1 nM, GSK2256294 reduced IL-1β-stimulated expression of *TNFα* by 38.56%, *IL1β* by 48.33%, *IL6* by 42.56%, and *IL8* by 44.91% in hMC (Fig. 5a–d. *p* < 0.0001, *p* < 0.0001, *p* < 0.0001, *p* < 0.0001, respectively). At 10 nM, GSK2256294 reduced IL-1β -stimulated expression of *TNFα* by 79.45%, *IL1β* by 79.96%, *IL6* by 62.26%, and *IL8* by 78.05% in hMC (Fig. 5a–d. *p* < 0.0001, *p* < 0.0001, *p* < 0.0001, *p* < 0.0001, respectively).

### 11,12-EET or 19,20-EDP reduces PA- and IL-1β-induced NFκB promoter activity

**Figure 6:**
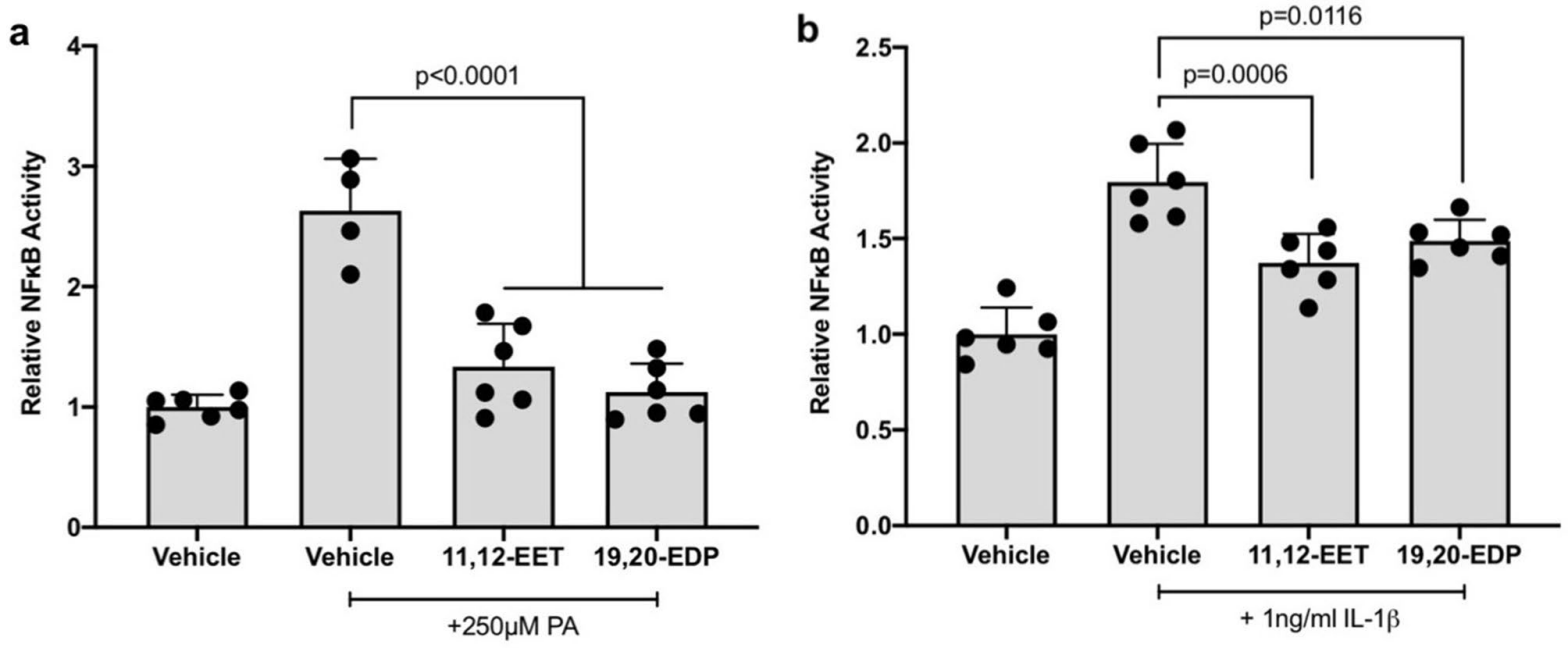
The effect of 11,12-EET or 19,20-EDP on PA or IL-1β-induced NFκB promoter activity. hMC were transfected with a NFκB-luciferase reporter construct and treated with (**a**) PA (**b**) IL-1β or in the presence or absence of 11,12-EET (0.5 μM) or 19,20-EDP (0.5 μM). NFκB activity was determined by measuring the ratio of firefly-to-renilla luciferase luminescence activity. Each bar represents the mean ± SD (n = 4, 5 or 6).

hMC were transfected with a NFκB-luciferase promoter-reporter construct and treated with PA or IL-1β in the presence or absence of 11,12-EET or 19,20-EDP. As shown in Fig. 6, 11,12-EET and 19,20-EDP decreased both PA- and IL-1β-induced NFκB-dependent luciferase activity. 11,12-EET and 19,20-EDP decreased PA-induced reporter activity by 49.2% and 57.3%, respectively (Fig. 6a. *p* < 0.0001, *p* < 0.0001), and they decreased IL-1β-induced reporter activity by 23.6% and 17.2%, respectively (Fig. 6b. *p* = 0.0006, *p* = 0.0116).

## Discussion

EET or EDP in combination with sEHi were previously shown to mitigate several DR-relevant experimental endpoints including: the expression of leukocyte adhesion proteins by hRMEC, peripheral blood monocyte (PBMC) adhesion to hRMEC monolayers, and TNFα-induced retinal leukostasis in mice^3^. In these studies, epoxygenated fatty acids were determined to act through NFκB-dependent signaling^3^. The anti-inflammatory potency of these lipid mediators in hRMEC caused speculation of their efficacy in other retinal cell types that are known to contribute to inflammatory conditions, such as glia. MC are potent propagators of preliminary inflammation and serve as a functional link between the neuronal and vascular compartments of the vertebrate retina^35^. MC span nearly the entire thickness of the retina and control retinal homeostasis including recycling neurotransmitters, maintaining the inner blood-retinal barrier, regulating retinal cation flux, and maintaining photoreceptor function^35^. MC function in innate immunity^36^ and some believe that diabetes-induced inflammation causes MC dysfunction, leading them to become destructive and promote DR pathogenesis^35^. Additionally, changes in MC have been observed prior to the appearance of overt vascular pathology in DR^35,37^. Consequently, therapeutics that block diabetes-related MC inflammation could prevent or slow the onset and progression of early DR.

11,12-EET and 19,20-EDP were selected for these experiments because both demonstrated efficacy in previous studies^3,38^. Additionally, 19,20-EDP is the most highly abundant regioisomer in the retina because CYP epoxygenases preferentially mono-oxygenate the terminal double bond of DHA, and sEH hydrolysis of 19,20-EDP is slower compared to the other regioisomers^19,39,40^. Therefore, 19,20-EDP turnover is presumably lower, enhancing efficacy through increased bioavailability. We chose 11,12-EET because it was one of the most abundant EET regioisomers in tissues and demonstrated potent anti-inflammatory activities in previous studies^38,39,41^. Notably, compared to other regioisomers, 11,12-EET and 19,20-EDP were also found in higher concentrations in MC-conditioned medium as determined by our mass spectrometric analysis (Supp. Fig. 1). It is important to note that EET and EDP are relatively unstable, and thus sEH inhibitors are a viable therapeutic route to increase epoxide levels and are currently under development for human use^28,42,43^. With the use of sEH inhibitors, all epoxygenated fatty acid regioisomers would be protected increasing their biological half-lives and activities. Notably, with the application of sEH inhibitors, any efficacy observed against inflammation presumably results from a summed response to all regioisomers, thus diminishing the significance of any single regioisomer’s contribution.

Before testing the effects of increasing epoxygenated fatty acids levels in hMC, we first investigated the effects of their depletion. CYP epoxygenase activity is responsible for converting AA and DHA to regioisomeric EETs and EDPs respectively^3^. hMC were treated with the broad-spectrum CYP inhibitor SKF-525a to reduce intracellular EET/EDP levels. In the presence of SKF-525a we observed significant increases in the expression levels of the DR-relevant inflammatory cytokines *TNFα*, *IL1β, IL6* and *IL8.* Others have shown that the proinflammatory effects of SKF-525a in cells are reversed by the addition of exogenous EETs, suggesting SKF-525a acts specifically by EET/EDP depletion^38,44,45^. Combined, these observations support our hypothesis that EET/EDP depletion, such as that occurring in DR, promotes hMC inflammation (Fig. 1). Exogenous addition of 11,12-EET, 19,20-EDP, and the sEH inhibitor GSK2256294, demonstrated a potent capacity to reduce inflammatory cytokine expression in hMC activated by PA and IL-1β. While previously demonstrated in hRMEC, this is the first report of the anti-inflammatory potential of these agents in retinal glia.

We hypothesize that in the earliest stages of DR pathogenesis, the predominant stimuli are those imposed by metabolic dysfunction such as elevated glucose and/or free fatty acids in the bloodstream and ocular tissues. Abnormal levels of glucose and/or FFA may cause damage to retinal cells that respond by producing and releasing inflammatory cytokines. These cytokines amplify through autocrine and paracrine mechanisms and become the dominant inflammatory stimulus in late-stage DR. Accordingly, we purposefully chose two stimuli, one from each of these stages, to test whether epoxides could continuously intervene, as the weights of these respective stimuli shift along the temporal axis of DR pathogenesis. Past experiments show that, among non-neuronal retinal cells, MC demonstrated the greatest increases in expression and secretion of inflammatory mediators in response to metabolic stimuli. Accordingly, we believe that MC act as the primary driving force of chronic inflammation in DR through their synthesis, release and auto-amplification of inflammatory cytokines that propagate inflammation in neighboring vascular and neuronal cells. While elevated glucose is commonly used to simulate diabetic conditions in vitro, we found that elevated glucose yields little to no response when studying many primary human retinal cells^8^. However, FFAs reliably and consistently induce inflammation in these cells consistent with DR^8^. Thus, we studied the response of primary human retinal cells to a free fatty acid, PA, that plausibly models the influence of diabetes-associated dyslipidemia^8^. We specifically demonstrated the effectiveness of PA as a DR-appropriate stimulus for human Müller cells. We chose to use 250 μM PA because it is physiologically relevant. Analysis of plasma free fatty acids determined PA to be at a concentration of 234.9 + /- 58.1 μmol/l in obese diabetic individuals fasted overnight^46^. Similar studies aiming to create comprehensive profiles of fatty acids in the plasma of type 2 diabetics have substantiated this finding^47^, and it is widely accepted that the lipid composition of peripheral tissues often reflect plasma levels. Furthermore, this concentration is within ranges used in studies of other retinal cell behaviors^48^, as well as other in vitro studies of diabetes^49,50^. We chose to use 1 ng/ml IL-1β empirically, because this concentration promoted elevated expression of TNFα, IL-1β, IL-6 and IL-8 in hMC cultures like that observed in the vitreous of diabetic patients and retina of experimental diabetes models^12,14,32,50–54^. Likewise, in vivo*,* cytokine-producing Müller cells are juxtaposed to vascular and neuronal responder cells, causing local concentrations at surface receptors that are higher than those measured in ocular fluids, retinal lysates and sera. Our chosen concentration of IL-1β is well within the range of those concentrations tested in several published studies^16,55^ mimicking cytokine amplification via autocrine and paracrine mechanisms. Finally, while reduced IL-1β concentrations could also be relevant to DR inflammation, EET/EDPs and sEH inhibition proved efficacious when tested against our model of severe inflammation induced by 1 ng/ml, suggesting efficacy of this therapeutic strategy over a range of inflammatory conditions that reflect DR onset and progression.

Our data demonstrate that exogenous administration of 11,12-EET and 19,20-EDP significantly decreased hMC cytokine expression induced by the two different inflammatory stimuli, PA and IL-1β. We also demonstrated that these epoxide-dependent activities manifest at the protein level in hMC when using experimental conditions that enhanced the levels and biological half-lives of the epoxides in culture (Supp. Fig. 2). While both epoxygenated fatty acids decreased PA-induced *TNFα* expression, they exacerbated IL-1β-induced *TNFα* expression, suggesting a different mechanism of action in the two cases. The exact mechanism of action by which EET and EDP function has yet to be determined, though the results of our NFκB-luciferase experiments indicated that both epoxygenated fatty acids decrease cytokine expression, at least in part, by modulating pathways that converge on NFκB-dependent transcription. NFκB is a pro-inflammatory transcription factor that controls the expression of inflammatory cytokines, and it plays an important, well recognized role in early DR pathogenesis^12^. Similar findings were obtained in our previous studies using human retinal microvascular endothelial cells, and there is ample precedent for this mechanism occurring in other cells and tissues^3,56^. Saturated fatty acids activate toll-like receptors expressed by MC that are upstream of NFκB-dependent transcription^57,58^. Additionally, the canonical IL-1β signaling pathway includes NFκB activation^5^. Therefore, we speculate that EET and EDP decrease *IL1β*, *IL6*, *IL8* and PA-induced *TNFα* mRNAs in part by an NFκB-dependent mechanism, while another signaling mechanism becomes overriding in the case of IL-1β-induced effects on *TNFα* mRNA. We do not consider this observation a deterrent to this therapeutic approach because we have previously shown that EET and EDP decrease TNFα-induced leukocyte adhesion functions in hRMEC^3^. Therefore, any potentially detrimental effects of MC-derived TNFα on the retinal endothelium would be mitigated downstream.

We also tested the capacity of sEH inhibitor GSK2256294 to reduce inflammatory cytokines in PA- and IL-1β-treated hMC. GSK2256294 blocks the hydrolysis of endogenous EET/EDP, raising their endogenous cellular concentrations to therapeutic levels. The results of several studies indicate that sEH inhibition is a promising therapeutic modality in a wide variety of systems. In our studies, we observed a consistent reduction of cytokine mRNAs across all GSK2256294 concentrations tested (0.1 nM, 1.0 nM, and 10 nM). Interestingly, while hMC responded to sEH inhibition alone, hRMEC do not, suggesting that hMC may be the main sight of bioactive sEH that affects paracrine EET/EDP. Similarly, others have shown that sEH is more highly expressed in MC compared to other retinal cells types^3,59^. While GSK2256294 potently inhibits sEH activity in HMC, it is important to note that it can exert off-target effects related to the end points explored in this study. For instance, sEH inhibition has been correlated with increased concentrations of lipoxin A4, an anti-inflammatory compound that resolved vascular damage and inflammation^60^. However, in the present study, this metabolite was not detected when queried in the conditioned medium of MC by mass spectrometric analysis. sEH is constitutively expressed in the retina and is elevated in diabetic murine retina, human retina and in human vitreous^3,59^. sEH activity in diabetes is thought to be responsible for pericyte loss and endothelial barrier dysfunction by promoting the production of pro-inflammatory diol 19,20-DHDP, the hydrolysis product of 19,20-EDP^59^. 19,20-DHDP alters the localization of cholesterol-binding proteins in the cell membrane, disrupting pericyte-endothelial cell junctions and inter-endothelial cell junctions^59^. Like the expression of sEH, the accumulation of 19,20-DHDP is significantly increased in samples from patients with diabetic retinopathy^59^. To ensure that potential activity from vicinal diols did not confound any of the cytokine measurements observed in our experiments, we treated hMC with 11,12-DHET and 19,20-DHDP. Neither lipid metabolite increased any of the inflammatory cytokines that were assayed in this study.

Mimicking a chronic, multifaceted disease like DR is a challenge in vitro, but in vitro experiments remain crucial tools to dissect the mechanisms of disease in a controlled, step-wise fashion. We used primary human Müller cells in order to maintain physiological relevance in our studies and to more easily translate our findings to future clinical trials in humans. Our proposed therapeutic strategy provides a unique advantage in translation to the clinic because it relies on manipulation of an endogenous system, allowing for protection throughout multiple stages of DR progression, while at the same time minimizing toxicity. Current mainstream therapies focus on mediating late-stage DR morbidities directly associated with vision loss, while herein we propose a strategy that would focus on chronic retinal inflammation in early-stage DR, before irreversible damage has commenced. Our results confirm the anti-inflammatory effects of epoxide elevation in hMC, paving the way for directed in vivo studies. In future studies, we hope to confirm the therapeutic potential of systemically administered epoxides over longer time spans of pathogenesis in in vivo models of DR. These studies will be enabled by the recent development of water-soluble analogues of the epoxygenated fatty acids, as they will overcome issues of hydrophobicity and turnover of the parent EET/EDPs, enhancing their systemic circulation and bioavailability^61^. In conclusion, our data indicate that therapeutic manipulations to increase retinal levels of epoxygenated fatty acids offer the potential to be highly efficacious in the treatment of DR.

## Methods

### Human Müller cell culture

Human tissue samples were obtained courtesy of the Advancing Sight Network, Birmingham Alabama. All experiments were approved and performed in accordance with guidelines by the Vanderbilt University Medical Center Institutional Biosafety Committee. Human Müller cells (hMC) were isolated from human donor tissue (NDRI, Philadelphia, PA, USA) within 24 h postmortem. The retinas were dissected from the eyecups and dissociated in Dulbecco’s modified Eagle’s medium (DMEM; Life Technologies; Carlsbad, CA) containing trypsin and collagenase (Worthington Biochemical Corp; Lakewood, NJ). Following incubation in dissociation medium, cells were grown in DMEM containing 10% fetal bovine serum (FBS) (R&D Systems; Minneapolis, MN) and 1X antibiotic/antimycotic solution (Thermo Fisher Scientific Asheville LLC; Asheville, NC). Cells were incubated at 37 °C, 5% CO_2_, 20.9% O_2_, and 95% relative humidity. Collectively, these conditions favor the survival of MC over other retinal cell types^62^. If needed, cultures were policed for removal of non-MC or colonies of pure MC were sub-cloned into a new dish. Final MC purity of cultures was > 97% and was determined by immunohistochemistry IHC with antibodies against cellular retinaldehyde-binding protein (CRALBP), glutathione synthetase (GS), and glial fibrillary acidic protein (GFAP). Passages 4 to 6 were used for all experiments.

### Human Müller cell treatment (SKF-525a, PA, IL-1β, 11,12-EET, 19,20-EDP, GSK2256294)

In preparation for treatment, hMC were seeded in 6-well dishes and grown to 70% confluence using 10% FBS-containing DMEM culture medium. Culture media were changed to serum-reduced conditions (2% FBS) for 12 h before treatment. Cells were treated with SKF-525a (5.0 μM; ENZO Life Science, Farmingdale, NY, USA) or vehicle for 24 h. *Experiments using PA as a stimulus are described as follows.* Cells were treated for 24 h in 2% FBS medium with BSA-bound palmitic acid (PA; 250 μM; Sigma-Aldrich; St Louis, MO) or fatty acid-free BSA vehicle (100 mg/ml in PBS; Sigma-Aldrich; St Louis, MO). BSA-bound PA was prepared by dissolving PA in EtOH at 200 mM. This PA/EtOH solution was mixed for 2 h at 37 °C with 100 mg/ml BSA in PBS to yield 5 mM PA before dilution to the final concentration of 250 μM in culture media. hMC treated with BSA-bound PA were co-treated during the final 3 h of the 24-h PA treatment with 0.1 nM, 1.0 nM, or 10 nM GSK2256294 (sEH inhibitor; Axon Medchem LLC; Reston, VA); 11,12-EET (0.5 μM; Cayman Chemical; Ann Arbor, MI); or 19,20-EDP (0.5 μM; Cayman Chemical; Ann Arbor, MI). *Experiments using IL1β as a stimulus are described as follows.* Cells were treated for 8 h in 2% FBS-containing DMEM culture medium supplemented with 1.0 ng/ml of human recombinant protein IL-1β (R&D Systems; Minneapolis, MN) and vehicle, 0.1 nM, 1.0 nM, or 10 nM GSK2256294; 11,12-EET (0.5 μM); or 19,20-EDP (0.5 μM). In experiments using GSK2256294, cells were pretreated with corresponding concentrations for 2 h before treatment with IL-1β. In all experiments, epoxygenated fatty acid concentrations (0.5 μM) were based on our previously published studies and literature precedents.

### Real-Time Quantitative Reverse Transcription PCR (qRT-PCR) of IL-1β, IL-6, IL-8 and TNFα mRNAs

After treatment, cells were washed twice with cold PBS, lysed with RNeasy Lysis Buffer (RLT; Qiagen; Germantown, MD), and total RNA was isolated using an RNeasy Mini kit (Qiagen; Germantown, MD). RNA was reverse transcribed to cDNA using the High-Capacity cDNA Archive Kit (Applied Biosystems; Waltham, MA). qRT-PCR was performed in duplicate by co-amplification of cDNA vs. *18S* using gene-specific TaqMan Gene Expression Assays (Applied Biosystems). The delta Ct method was used to determine relative expression of the targeted mRNA normalized to 18S levels. These commercial assays were performed according to the manufacturer’s protocol.

### NFκB promoter assay

hMC were seeded on 96-well black-walled, clear bottom plates. Each well was transfected with NFκB-luciferase promoter-reporter, negative control, or positive control constructs, from the Cignal NFκB Reporter Assay (Qiagen). Seventy-five μL of fresh 10% medium was added to each well 30 min prior to transfection. A transfection mixture was prepared in a separate PCR tube, consisting of 200 ng of construct, 1.8μL of Targefect solution A (Targeting Systems; El Cajon, CA), and 3.6μL Virofect (Targeting Systems) in 50μL of Optimem (Life Technologies). Fifteen tube inversions were performed between the additions of each reagent, and the transfection mixture was incubated at 37 °C for 25 min before use. Fifty μL of the transfection mixture was added per well of cultured hMC. Twelve hours after transfection, cells were washed and treated with fresh 10% medium for 12 h. Twenty-four hours post-transfection, cells were treated with vehicle, IL-1β (1.0 ng/ml) or PA-BSA (250 μM) in the presence or absence of 11,12-EET (0.5 μM) or 19,20-EDP (0.5 μM) for 4 h and 8 h respectively. Luciferase activity was quantified using the Dual-Glo Luciferase Assay System (Promega; Madison, WI), according to the manufacturer’s protocol. Data are reported as the relative ratio of firefly-to-renilla luciferase.

### Statistical analysis

Data were analyzed using Prism software (GraphPad; La Jolla, CA). T Test and ANOVA with Tukey’s multiple comparisons post-hoc test were used to evaluate significant differences among treatment groups. Values of *p* < 0.05 were considered statistically significant.

### Consent for publication

All authors consent for publication.

## Supplementary Information

Supplementary Information 1.

## Data Availability

Data and materials will be available upon request.
