# Author Correction: Cytochrome P450-epoxygenated fatty acids inhibit Müller glial inflammation

**DOI:** 10.1038/s41598-021-98425-7

**Published:** 2021-09-16

**Authors:** Cayla D. Ontko, Megan E. Capozzi, Minjae J. Kim, Gary W. McCollum, John S. Penn

**Affiliations:** 1 Department of Molecular Physiology and Biophysics, Vanderbilt University School of Medicine, Nashville, TN USA; 2 Duke Molecular Physiology Institute, Duke University, Durham, NC USA; 3 Department of Ophthalmology and Visual Sciences, Vanderbilt University Medical Center, Nashville, TN USA

Correction to: *Scientific Reports* https://doi.org/10.1038/s41598-021-89000-1, published online 06 May 2021

The Supplementary Information file published with this Article contained errors.

In the Supplementary Figure 2, the scale of the y-axis in Panel (a) was incorrect.

Furthermore, under the subheading ‘Soluble Protein Quantification Method’,

“Secreted protein concentrations are reported as pg/mL/µg total cellular protein.”

now reads:

“Secreted protein concentrations are reported as pg/mL.”

The original Supplementary Information 1 file is provided below.

These errors have now been corrected in the Supplementary Information 1 file that accompanies the original Article.

## Supplementary Information

Supplementary Information 1.

