# Supplementary material for: Cytochrome P450-epoxygenated fatty acids inhibit Müller glial inflammation
Source: Sci Rep. 2021 May 6;11:9677. doi: 10.1038/s41598-021-89000-1 (PMC8102485; doi:10.1038/s41598-021-89000-1)
Supplement: Supplementary file 1 — Supplementary Information 1. [file 41598_2021_89000_MOESM1_ESM.pdf]

Supplementary Information

Cytochrome P450-epoxygenated fatty acids inhibit Müller glial inflammation

Supplementary Information:

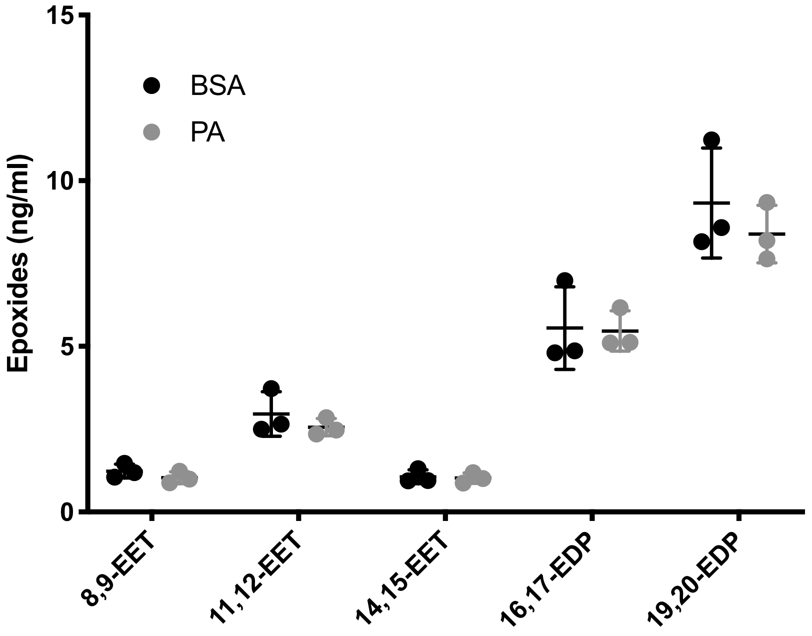

Supplementary Figure 1: The effect of PA on EET/EDP levels in Müller cell culture media. Müller cells were treated with either BSA (vehicle; 100mg/ml) or BSA-conjugated PA (250μM) for 24 hours. AA (10μM) or DHA (10μM) was added for the last 3 hours of treatment. PA had no effect on epoxide levels in this preliminary experiment (n=3).

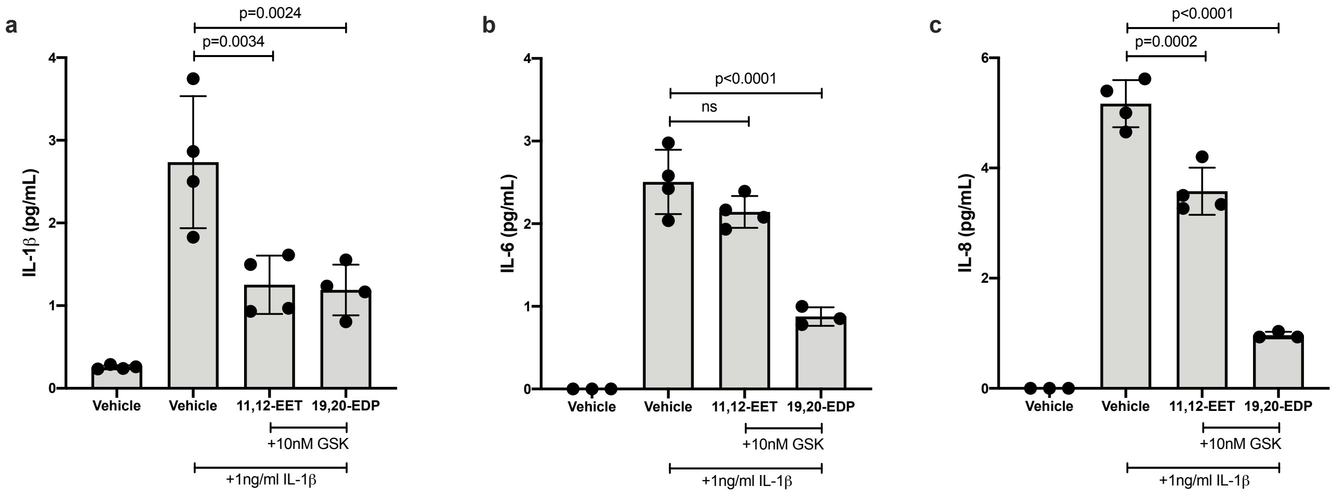

Supplementary Figure 2: The effect of 11,12-EET or 19,20-EDP and GSK on IL-1β-induced inflammatory mediator secretion in Müller cells. Human Müller cells were pretreated with 11,12-EET or 19,20-EDP (0.5µM) and GSK2256294 (10nM; sEH inhibitor) for 2 hours before IL-1β (1ng/ml) was added for 8 hours. The media were changed to remove IL-1β, and 11,12-EET or 19,20-EDP and GSK2256294 were added at the same concentrations for another 8 hours. Cytokine secretion was assayed by (a) ELISA and (b, c) AlphaLISA. (a) IL-1β, (b) IL-6, and (c) IL-8 secretion was significantly decreased with the use of epoxygenated fatty acid and sEH inhibitor. Bars represent mean ± SD (n=3-4). Two outliers removed with Grubbs Test.

Soluble Protein Quantification Method:

HMC were seeded in 24-well plates and grown to 70% confluence using 10% FBS-containing DMEM culture medium. Culture media were changed to serum-reduced conditions (2% FBS) for 12 hours before treatment. Cells were pretreated for 2 hours with GSK2256294 (10nM; Axon Medchem LLC; Reston, VA) and 11,12-EET (0.5µM; Cayman Chemical; Ann Arbor, MI) or 19,20-EDP (0.5µM; Cayman Chemical). Media were removed and cells were treated with media containing IL-1β (1ng/ml; R&D Systems; Minneapolis, MN), GSK2256294 and 11,12-EET or 19,20-EDP for 8 hours. Media were removed to remove IL-1β and cells were treated with GSK2256294 and 11,12-EET or 19,20-EDP again for 8 hours. After treatment, culture media were collected and assayed for IL-1β, IL-6, and IL-8 using the IL-1 beta Human ELISA Kit, High Sensitivity (Thermo Fischer Scientific, Waltham, MA) and IL-6 or IL-8 (human) AlphaLISA Detection Kit (PerkinElmer, Inc., Waltham, MA). Cells were washed with cold PBS and lysed using RIPA buffer (Thermo Fischer Scientific), and the protein concentration of cell lysates was determined using a bicinchoninic acid assay (Pierce; Rockford, IL). Secreted protein concentrations are reported as pg/mL.
